# Effect of Oxidative Stress on Reproduction and Development

**DOI:** 10.3390/antiox11020312

**Published:** 2022-02-03

**Authors:** Giulia Guerriero, Gerardino D’Errico

**Affiliations:** 1 Department of Biology, University of Naples Federico II, Complesso Universitario di Monte S. Angelo, via Cinthia 6, 80126 Naples, Italy; 2 Department of Chemical Sciences, University of Naples Federico II, Complesso Universitario di Monte S. Angelo, via Cinthia 4, 80126 Naples, Italy

There is a growing amount of literature on the effects of oxidative stress resulting from the imbalance between prooxidants and antioxidants [1,2]. Stressors, by inducing physiological and reproductive disorders, determine failures in various cellular processes such as embryogenesis, development, differentiation and growth, threating the survival of the living species [3,4,5,6]. Although a definite role of free radicals and antioxidants is well established, there is sparse knowledge of their role in a multitude of stressors such as temperature fluctuations, osmotic stress, alterations in oxygen availability and other anthropogenic impacts, all factors which can directly affect free radical chemistry during reproduction and development. The Special Issue “Effect of Oxidative Stress on Reproduction and Development” has been conceived to set out the knowledge on biodiversity conservation and sustainability. It also deals with a new approach to oxidants and/or antioxidants detection. Here, we offer an overview of the contents of this Special Issue, which collects eight original articles and two reviews.

Various studies show how the insults of oxidative stress can be contrasted using natural substances [7,8,9,10]. In particular, Mottola et al. analyze the DNA protection provided by the potential therapeutic applications of substances of natural origin such as α-lipoic acid (LA) and curcumin (Cur), against hydrogen peroxide (H_2_O_2_)-induced damage in human amniotic cells in vitro [7]. The results highlighted that there is a protective action of LA against oxidative DNA damage. Similarly, Cur alone is able to reduce the DNA fragmentation index. Interestingly, the combination of LA and Cur delivers a valid antigenotoxic effect. Sun and coworkers studied the role of N-acetyl-L-cysteine (NAC) in the developmental competence of bovine oocytes and embryos cultured in vitro and concluded that NAC affects early embryonic development, in a dose-dependent and stage-specific manner [8]. Its supplementation has beneficial effects through the prevention of apoptosis and the elimination of oxygen free radicals during maturation and culture in vitro. The authors suggest NAC supplement as a preventive antioxidant for in vitro embryo production. Ab Hamid et al. show that royal jelly effectively contrasts imbalances in the reproductive system [9]. Particularly, royal jelly supplementation in rats affected by polycystic ovarian syndrome improves reproductive parameters, leading to the recovery of various stages of ovarian follicular development due to its anti-androgenic effect through antioxidant action and to the modulation of estrogenic activity. This is quantitatively detected as a high follicle-stimulating hormone level, and low luteinizing hormone, testosterone and estradiol levels. De Luca et al. review the correlation between oxidative stress and male fertility and on the specific role of antioxidants and inositols [10]. Oxidative stress is a common reason for several conditions associated with male infertility. High levels of reactive oxygen species (ROS) impair sperm quality by decreasing motility and increasing the oxidation of DNA, protein and lipids. Multi-antioxidant supplementation is considered effective for male fertility parameters. In addition, other natural molecules such as myo-inositol (MI) and d-chiro–inositol (DCI) ameliorate sperm quality.

The work of Jung et al. focused on diabetic embryopathy susceptibility [11]. The results indicate that murine embryonic stem cell lines, which are embryopathy-susceptible, are more dependent on exogenous glucosamine for glycosylation, stimulation of the pentose phosphate pathway and ADPH production than embryopathy-resistant embryos. Furthermore, embryopathy-susceptible embryos are more prone to induce oxidative stress by high glucose levels. Alcala et al. report an interesting study on the effect of oxidative stress in obesity-induced teratogenesis [12]. The results show that pregestational obesity in rats induces hepatic protein and DNA oxidation and a decline in antioxidant enzymes. Particularly, obesity causes malformations through the depletion of maternal glutathione, thereby decreasing glutathione-dependent free radical scavenging in embryos, which can be prevented by vitamin E supplementation.

Special attention has also been paid to the toxic mechanisms of action with pleiotropic outcomes on reproduction and to the technical approach useful to evaluate the decline in sperm motility via oxidative damage [13,14]. The review by Meli et al. summarizes data from several oxidative studies demonstrating how the induction of oxidative stress may contribute to the reproductive adverse effects observed after BPA exposure across animal species and humans [13]. This review described the protective effects of five classes of antioxidants—vitamins and co-factors, natural products (herbals and phytochemicals), melatonin, selenium and methyl donors (used alone or in combination)—that have been reported useful to counteract BPA reprotoxicity. In another original research study related to sperm quality assessment, oxidative stress and mitochondrial activity, Gallo et al. explored for the first time their correlation in two marine invertebrates, *Ciona robusta* and *Mytilus galloprovincialis*, and one mammalian, *Bos taurus*, sperms [14]. They concluded that energy sources for sperm motility vary between species and that ROS causes a decline in sperm motility via oxidative damage of membrane lipids. Furthermore, this study validated the fluorescent probes used in combination with a spectrofluorometer as a simple and powerful methodology for supplementary sperm quality evaluation.

Two studies focus on the oxidative stress resulting from exposure of women to tobacco smoke during pregnancy [15,16]. Bizon et al. revealed altered levels of copper, cadmium and zinc in the blood [15]. Metallothionein, superoxide dismutase and glutathione peroxidase were found to be the important antioxidants during early pregnancy, when exposure to tobacco smoke occurs. Moreover, women’s exposure to cigarette smoking potentially affects the endometrium, as discussed by Kida et al. [16]. These researchers showed that an extract of cigarette smoke increases reactive oxygen species levels and stimulates the hypoxia-inducible factor (HIF)-1 in primary human endometrial stromal cells. The results indicated that HIF-1 might play an important regulatory role in cellular stress, inflammation and remodeling induced by exposure to cigarette smoke.

All of the articles in this Special Issue demonstrate that establishing a better understanding of oxidative stress is of pivotal importance in reproduction and development. The variety of subjects treated proves that this is a complex and multifaceted topic on which researchers are working from different viewpoints and perspectives. We thank all the authors for their contributions. We hope that this Special Issue will encourage young, as well experienced, scientists to move forward on the path to increasing knowledge in this research area.
